# Protection by natural cholera against later episodes of cholera over 10 years of follow-up in Matlab, Bangladesh: a retrospective cohort study

**DOI:** 10.1016/j.lanmic.2024.100981

**Published:** 2025-03

**Authors:** Masuma Hoque, Deok Ryun Kim, Faisal Ahmmed, Md Taufiqul Islam, Justin Im, Birkneh Tilahun Tadesse, Sophie Kang, Farhana Khanam, Fahima Chowdhury, Tasnuva Ahmed, Md Golam Firoj, Asma Binte Aziz, Suman Kanungo, Ashraful Islam Khan, Md Alfazal Khan, Jooah Lee, Gideok Pak, Florian Marks, Jerome H Kim, Andrew Azman, Jan Holmgren, Firdausi Qadri, Khalequ Zaman, John D Clemens

**Affiliations:** a International Centre for Diarrhoeal Disease Research, Bangladesh, Dhaka, Bangladesh; b International Vaccine Institute, Seoul, South Korea; c National Institute of Cholera and Enteric Diseases, Kolkata, India; d Cambridge Institute of Therapeutic Immunology and Infectious Disease, Department of Medicine, University of Cambridge School of Clinical Medicine, Cambridge, UK; e Bloomberg School of Public Health, Johns Hopkins University, Baltimore, MD, USA; f Geneva Centre for Emerging Viral Diseases, Geneva University Hospitals, Geneva, Switzerland; g Division of Tropical and Humanitarian Medicine, Geneva University Hospitals, Geneva, Switzerland; h Department of Microbiology and Immunology, University of Goteborg, Goteborg, Sweden; i UCLA Fielding School of Public Health, Los Angeles, CA, USA; j Vaccine Innovation Center, Korea University School of Medicine, Seoul, South Korea

## Abstract

**Background:**

Patients with cholera have been shown to be protected against subsequent cholera for 3 years after their initial episode. We aimed to assess protection at 10 years of follow-up.

**Methods:**

In this retrospective cohort study, cohorts of patients treated for cholera (index patients) and contemporaneously selected age-matched individuals without cholera (controls), randomly selected from the population of Matlab, Bangladesh, were assembled between 1990 and 2009 and followed for up to 10 years. Selection of participants who had no history of cholera in the 5 years before selection proceeded in secular sequence, and selection was done without replacement. Protection against subsequent treated cholera was assessed in proportional hazards models and waning of protection was assessed non-parametrically with use of smoothing of protection curves.

**Findings:**

We included 3925 index patients and 23 550 matched controls. Patients with El Tor cholera (26 subsequent episodes among 3619 index patients) had a 48·6% (95% CI 23·1 to 65·7; p=0·0012) lower risk of El Tor cholera than controls, with no evidence of waning during up to 10 years of follow-up (p=0·87). Index patients aged 5 years and older with El Tor cholera (nine subsequent episodes among 2279 index patients) were at a 61·7% (23·6 to 80·8; p*=*0·0065) lower risk of El Tor cholera, whereas index patients younger than 5 years with El Tor cholera (17 subsequent episodes among 1340 index patients) had a 36·2% (–5·0 to 61·3; p=0·077) lower risk (p=0·26 for the difference by age).

**Interpretation:**

Protection against El Tor cholera associated with previous El Tor cholera was moderate in magnitude and sustained over 10 years of follow-up. These findings suggest the potential for sustained, long-term protection by oral cholera vaccines in populations with endemic cholera and help inform models of cholera in endemic settings.

**Funding:**

Bill & Melinda Gates Foundation.

## Introduction

Cholera is an acute diarrhoeal disease caused by *Vibrio cholerae* bacteria belonging to the O1 and O139 serogroups. It is responsible for approximately 100 000 deaths annually and is the source of major disruptive epidemics among the world’s most vulnerable populations.^1^ Oral cholera vaccines (OCVs) consisting of inactivated antigens are safe and protective, and are deployed as part of a global OCV stockpile funded by Gavi, the Vaccine Alliance and coordinated by WHO. More than 40 million doses of OCV are now released annually.^2^

**Research in context**

**Evidence before this study**

To research the previously published literature on protection associated with an initial natural cholera episode against a later occurring cholera episode, we searched PubMed for studies using the search terms: “cholera”, “recurrent”, “natural”, “immunity”, “protection”, “endemic setting”, and “oral cholera vaccine” in the title, abstract, and keywords. We included studies in English published between Jan 1, 1966, and Dec 31, 2022. Studies were included if they focused on the protection by cholera episodes of sufficient severity to prompt the patient to seek treatment against later episodes of treated cholera in a population with endemic cholera. We identified four studies, all conducted in present day Bangladesh, of which three focused on initial and later cholera episodes among patients seeking treatment. These three studies each found protection associated with an initial episode, although no study followed these patients for more than 3 years. The one study with adequate power to evaluate protection by age at the initial cholera episode found similar protection for young children and for older individuals.

**Added value of this study**

We analysed clinical and microbiological data from a large, well studied population in rural Bangladesh followed for decades with comprehensive surveillance for treated cholera episodes and demographic events. When compared with contemporaneously selected individuals without cholera from the population, individuals with initial treated episodes of El Tor cholera had a moderate reduction in the risk of subsequent treated El Tor cholera, and this protection was sustained over 10 years of follow-up. This sustained reduction of risk was seen for individuals whose initial cholera episode occurred at ages below 5 years as well as at older ages, although the estimate of the reduction of risk was somewhat lower and not statistically significant for the younger age group.

**Implications of all the available evidence**

Our findings indicate that protection associated with natural El Tor cholera among patients seeking treatment in populations with endemic cholera can be sustained for very long periods, regardless of the age at the initial episode, probably due to the combination of immune responses induced by the initial infection and subsequent immunological boosting by subsequent infections. Overall protection against El Tor cholera associated with earlier El Tor cholera was moderate in magnitude, and protection associated with episodes in young children was suggestively lower than that for older individuals, patterns seen with currently licensed, inactivated oral cholera vaccines. These observations suggest that, unless future vaccines can elicit greater protective immunity against cholera than that provided by natural cholera, the upper limit of protection attainable with these vaccines might be moderate in magnitude, although as demonstrated elsewhere, their population-level impact can be greatly augmented by vaccine herd protection.

The focus on OCVs over the past decades has in part been motivated by studies showing that human *V cholerae* infections, either naturally occurring or experimentally induced, are immunising and confer protection against subsequent cholera.^3^, ^4^, ^5^, ^6^ Such studies done in populations with endemic cholera have demonstrated partial protection associated with previous natural cholera, but follow-up has been limited to only up to 3 years after initial episodes. Similarly, long-term follow-up of the pivotal field trial for one of the inactivated OCVs now used in the stockpile, Shanchol (Shantha Biotechnics, Hyderabad, India), has demonstrated sustained protection against cholera at 5 years of follow-up, and the outer limit of duration of protection is not known.^7^

Knowledge of the duration of protection associated with natural *V cholerae* infections can help inform expectations of current and future OCVs and is needed for epidemiological models used to predict trends of cholera incidence and to design OCV programmes. Here, we report on an analysis of long-term protection associated with treated, natural cholera episodes against subsequent episodes in a well studied population in rural Bangladesh with endemic cholera for which comprehensive surveillance for cholera has been undertaken for more than four decades.

## Methods

### Study design and participants

This retrospective cohort study was conducted in Matlab, a rural research site operated by the International Centre for Diarrhoeal Disease Research, Bangladesh (icddr,b) with a current population of approximately 240 000 individuals.^8^ This population has been documented to experience cholera outbreaks yearly, typically with surges of cholera in the pre-monsoon (March–June) and post-monsoon (July–September) seasons. The icddr,b provides the only source of care for diarrhoea occurring in the population, which is freely available. Systematic and comprehensive clinical and microbiological surveillance for cholera among all diarrhoea cases presenting for care has been conducted and entered into electronic data files since 1983. In addition, icddr,b has continuously monitored all births, deaths, and migrations in the population since 1966 through the Matlab Health and Demographic Surveillance System (HDSS).^8^ This computerised system allows for a day-by-day listing and characterisation of all people in the study area. Detailed methods for data collection and curation of the Matlab HDSS are published elsewhere.^9^ This combination of demographic and cholera surveillance enables evaluation of the occurrence of cholera in well studied cohorts of individuals with and without cholera to assess protection against subsequent cholera, an approach that has been used in past studies in Matlab.^3^, ^4^, ^5^ Using an approach similar to a previously published analysis,^5^ the general strategy was to assemble cohorts of patients treated for cholera in the surveillance (index patients) and of age-matched, contemporaneously selected individuals without cholera (controls). We then followed the cohorts for up to 10 years to assess the comparative rates of subsequent treated episodes of cholera, allowing for calculation of the protection associated with the initial treated cholera episodes. Ethical approval for this retrospective analysis was obtained from the Ethical Review Committee of icddr,b.

### Procedures

We analysed clinical and microbiological surveillance data from clinical records for all patients with diarrhoea presenting for care at the Matlab treatment centres operated by icddr,b. All patients presenting with diarrhoea routinely receive faecal cultures, which are evaluated in the Matlab station laboratories for *V cholerae* by serogroup, biotype, and serotype, using standard methods.^5^^,^^10^^,^^11^ Data for all diarrhoea episodes during the 5 years before the inception of selection of study participants (Jan 1, 1990) until the end of the follow-up (Dec 31, 2014) were assembled. Identification variables enabled linkage of patient information with the Matlab HDSS for additional information on potentially confounding variables (age, sex, religion*,* geographical location of residence). These data as well as data on clinical features and microbiological results were entered onto a structured data form, and then into a computerised database, with suitable procedures for data cleaning, including range, consistency, and completeness checks. The Matlab HDSS was also used to select and to obtain information on the same identifying and potentially confounding variables for the controls. Patients seen in the Matlab diarrhoeal surveillance were considered to have a cholera visit if, upon presentation to the treatment centre, they gave a history of diarrhoea and a faecal culture yielded *V cholerae* O1. Because patients with diarrhoea might report back to the treatment setting for further care of the same episode after being discharged, we grouped diarrhoea visits into the same episode if the date of presentation for a visit was within 7 days of discharge for the previous visit. A cholera episode was defined as a diarrhoeal episode for which a faecal culture yielded *V cholerae* O1. The date of selection of index patients was the initial date of presentation for care of the cholera episode; this date of selection also served as the date of selection for non-cholera controls matched to each case. For our analyses, we did not select index patients with O139 cholera because earlier studies had convincingly shown no cross-protection between infections by the O1 and O139 serogroups and because O139 has been rarely detected globally since the 1990s.^5^

To create a cohort of index patients with cholera, we first arranged in temporal order (oldest to most recent) cholera episodes that had dates of selection between Jan 1, 1985, and Dec 31, 2009. Then, beginning with dates of selection starting at Jan 1, 1990, we proceeded forward in time to sequentially select as index patients those who had resided in Matlab for at least 5 years before the date of selection and had no history of cholera in the 5 years before selection. For children younger than 5 years on the date of selection, we required that they were born in Matlab, had resided there since birth, and had no history of cholera since birth. Excluded from the index patient cohort were patients with cholera earlier selected as a cholera index patient or control. We started selection of index patients in 1990 because the previous cholera vaccine trial done in Matlab, a trial of inactivated OCVs, had been initiated in 1985 and no vaccine protection was evident after 3 years of follow-up.^12^ No cholera vaccine trials have been undertaken in Matlab subsequently.

To assemble the controls matched to the index patients, we arranged the selected index patients in temporal order from oldest to most recent, and sequentially and randomly selected six controls from the entire Matlab population for each index patient, matched by exact age on the date of selection, and with the additional requirements that the control did not live in the same *bari* (immediate neighbourhood) as the matched index patient, had to have lived in Matlab for at least 5 years before the date of selection and had no history of cholera during this time (or, if younger than 5 years on the date of selection, was born in Matlab, resided there since birth, and had no history of cholera since birth). A person selected as an index patient or a control was not eligible to be selected subsequently as an index patient or a control.

To assess the comparative rates of subsequent treated episodes of cholera and enable calculation of the protection associated with the initial treated cholera episodes, cohorts were followed for up to 10 years. Follow-up for estimation of rates started on the date of selection, and continued to the date of presentation for cholera, migration out of the study, death, or the end of study follow-up (Dec 31, 2014), whichever came first.

### Outcomes

The primary outcome was occurrence of an incident, treated episode of cholera following the date of selection. Secondary outcomes considered incident, subsequent episodes by biotype and serotype of *V cholerae* O1.

### Statistical analysis

Bivariate comparisons of cholera index patients and controls for categorical variables and dimensional variables were evaluated with χ^2^ tests and Student’s *t* tests, respectively. We assessed the protective effect (PE) associated with an index O1 serogroup cholera episode against a subsequent serogroup O1 cholera episode by comparing the post-selection hazard rates of cholera between the index patients and matched controls. Protection was expressed as:

$$\text{PE}=\left(1\;-\;\frac{\mathrm{Hazard}\;\mathrm{rate}\;\mathrm{in}\;\mathrm{index}\;\mathrm{cases}}{\mathrm{Hazard}\;\mathrm{rate}\;\mathrm{in}\;\mathrm{matched}\;\mathrm{controls}}\right)\;\times \;100$$

In unadjusted analyses, we calculated incidence density ratios and estimated p values with appropriate χ^2^ tests and 95% CIs, using test-based methods.^13^

We fitted Cox proportional hazards models with a robust sandwich covariance matrix estimate to account for the intracluster dependence between index patients and their matched controls, taking time to the date of presentation for the first episode of cholera occurring after the date of selection as the dependent variable.^14^ Several baseline variables, including age, sex, religion, distance of residence from the nearest river, and distance of residence to the nearest icddr,b diarrhoeal treatment centre were ascertained for all index patients and controls. To adjust estimates of PE for these potentially confounding covariates, we calculated hazard ratios in the Cox proportional hazards models, in which the dependent variable was time to the date of presentation for the first episode of cholera occurring after the date of selection, and independent variables included the cohort group variable (index patients *vs* controls) and the matching variable (age) as forced variables, as well as other baseline variables selected from these potentially confounding variables using forward stepwise models. Variables were selected for these models after verifying that the proportionality assumption using weighted Schoenfeld residuals was fulfilled, that their p value for entry was less than 0·1, and that their p value for retention was less than 0·05.^15^ Two-way interaction terms between stratifying variables and the variable for cohort group variable were introduced in these models to assess differences in protective associations by levels of strata. We performed a goodness of fit test that tests the joint significance of all variables in the model and provided the likelihood score test statistics along with the p value. In these models, the protection against a subsequent cholera episode associated with index cholera episodes was calculated from 1 – hazard ratio, where the hazard ratio was estimated by exponentiating the coefficient for cohort group variable, and the standard error of this coefficient was used to estimate p values and 95% CIs for the hazard ratio.^16^ Estimates from the models included only individuals with complete data on all variables pertaining to a particular analysis.

To analyse waning of PE, we fitted non-parametric proportional hazard models, using smoothing scaled Schoenfeld residuals from the models, as described elsewhere.^15^^,^^16^ A smoothed sum of the estimated proportional hazards coefficient and the scaled Schoenfeld residuals over time was used to estimate the functional form of the time-varying coefficient for the cohort group variable. The optimal smoothing parameter was selected from the fit of the LOESS (locally estimated scatterplot smoothing) curve by minimising the bias-corrected Akaike information criterion (AICC) that strikes a balance between the residual sum of squares and the complexity of the fit.^17^ The AICC was selected to overcome an undersmoothing tendency for small sample sizes. A zero slope indicated that the coefficient did not vary with time, and the corresponding p value, derived from the statistical test of non-parametric proportional hazard for the group variable, gave the probability of departure from the zero slope and hence of waning of protection. P values were interpreted in a two-tailed fashion, and those less than 0·05 were considered significant. Statistical analyses were performed using SAS software, version 9.4 of the SAS System for Windows.

### Role of the funding source

The funder of the study had no role in study design, data collection, data analysis, data interpretation, or writing of the report.

## Results

**Figure 1:**
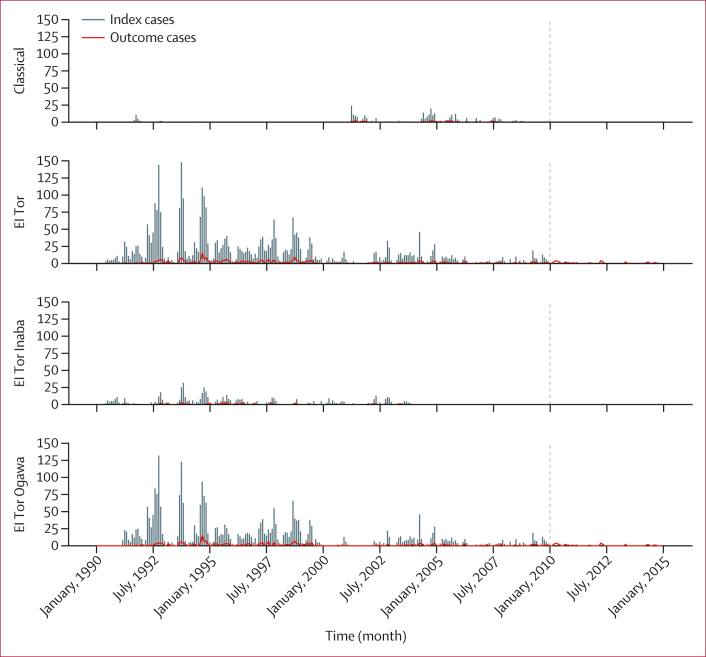
Cholera during the study period by biotype and serotype Monthly cholera cases are presented by biotype and serotype. Index patients selected between January, 1990, and December, 2009, are presented in the needle plot (grey). The dotted vertical line shows the end date of the index patient selection period. Cholera episodes detected during follow-up of index patients and controls between January, 1990, and December, 2014, are presented in the series line (red).

**Figure 2:**
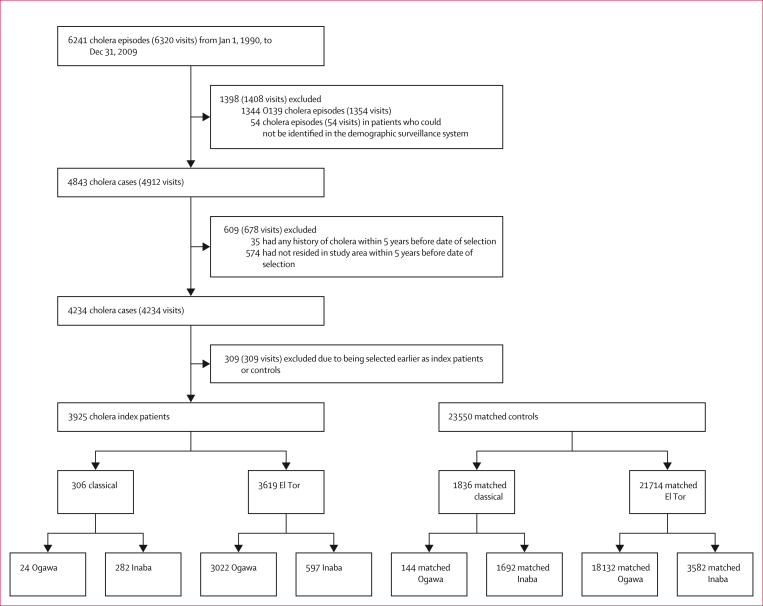
Assembly of index patients and controls

We included 3925 index patients and 23 550 age-matched controls during the period 1990–2009. 1445 (36·8%) index patients were children younger than 5 years. El Tor was the dominant strain (3619 [92·2%]), and 3022 (83·5%) of El Tor isolates had Ogawa serotype. 302 (1·1%) cholera episodes were detected after selection of index patients and controls during up to 10 years of follow-up. Figure 1 shows the incidence of cholera in Matlab between Jan 1, 1990, and Dec 31, 2014, the period of assembly and follow-up of cholera index patients, showing declining incidence over the study period. The assembly of the index patients for analysis is shown in figure 2.

**Figure 3:**
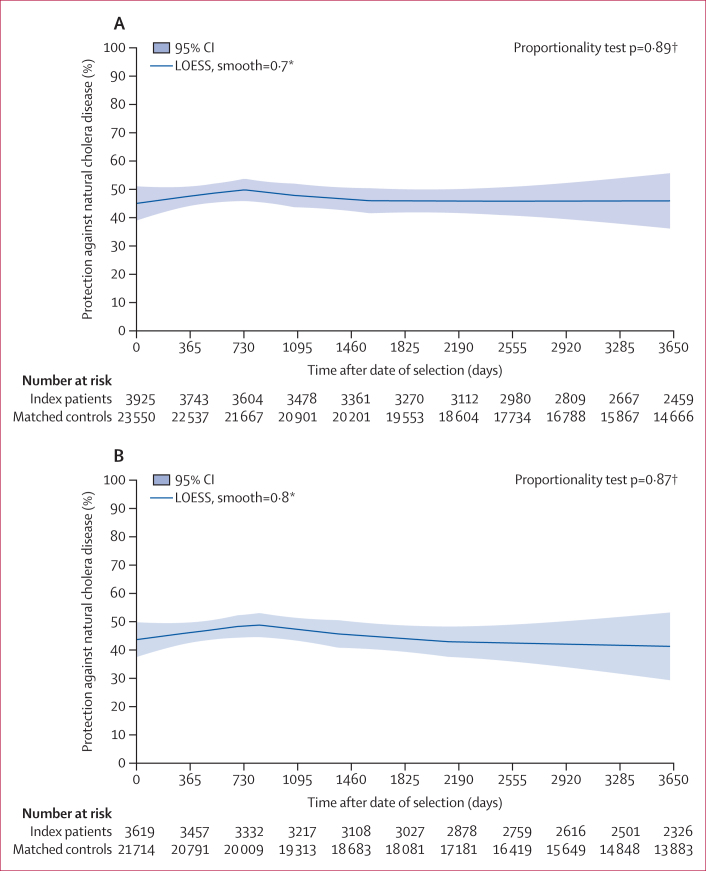
Smoothed curves for adjusted instantaneous protective associations between index and subsequent cholera for all episodes (A) and El Tor episodes (B) Associations were adjusted for the matching variable (age at selection), as well as selected demographic variables known to be associated with the risk of treated cholera in Matlab, including distance from residence to the Dhonagoda River and distance from residence to hospital. ∗LOESS (locally estimated scatterplot smoothing), a non-parametric technique that uses local weighted regression to fit a smooth curve through points in a scatter plot, is described in the Methods. Smooth=0·7 (A) and 0·8 (B), which are optimally chosen values for the smoothing parameter, which ranges from 0·1 to 0·9 using the bias-corrected Akaike information criterion. The figure presents the fitted localised regression curve using 70% (A) and 80% (B) neighbouring data points. †Proportionality test for waning of protection as described in the Methods.

The baseline characteristics of individuals and households were generally well balanced between the index patient and control cohorts, although index cases lived closer to a major river as well as to the nearest diarrhoeal treatment centre (table 1). As shown in table 2, during 10 years of follow-up, 29 post-selection episodes of cholera were observed among 3925 index patients, irrespective of biotype (0·25 episodes per 100 000 person-days) and 273 incident episodes were detected among 23 550 matched controls (0·39 episodes per 100 000 person-days; unadjusted PE 36·2% [95% CI 6·8–56·3], p=0·020; adjusted PE 50·2% [27·0–66·0], p=0·0004). The smoothed curves for instantaneous, adjusted protection showed less than a 5% drop in protection over 10 years, a decrease that was not significant (p=0·89; figure 3A).

**Table 1:** Characteristics of people with cholera (index patients) and age-matched people without cholera (matched controls) on the date of selection

	Index patients (n=3925)	Matched controls (n=23 550)	P value∗
Age <5 years at selection	1445 (36·8%)	8670 (36·8%)	1·00
Age at selection, years	16·5 (19·3)	16·5 (19·3)	1·00
Male	2015 (51·3%)	11 656 (49·5%)	0·033
Muslim	3604 (91·8%)	20 583 (87·4%)	<0·0001
Residence within 500 metres from Dhonagoda River	957 (24·4%)	3904 (16·6%)	<0·0001
Distance from residence to hospital, kilometres	4·72 (2·6)	6·84 (4·0)	<0·0001

Data are n (%) or mean (SD).

∗ P values were derived using the χ^2^ test for categorical variables and the *t* test for continuous variables.

**Table 2:** Protective associations between initial and recurrent cholera episodes overall, for El Tor biotype, and for El Tor biotype by serotype and age group at selection during 10 years of follow-up

	Participants, n	Cholera episodes during follow-up, n	Person-days follow-up, n	Incidence, per 100 000 person-days	Unadjusted protection, % (95% CI, p value)	Adjusted protection, % (95% CI, p value)∗
**Overall**						
All cholera outcomes						
All cholera index cases	3925	29	11 747 358	0·25	36·2% (6·8 to 56·3, p=0·020)	50·2% (27·0 to 66·0, p=0·0004)†
Matched controls	23 550	273	70 369 080	0·39	Ref	Ref
El Tor cholera outcomes						
El Tor index cases	3619	26	10 893 156	0·24	33·6% (0·8 to 55·5; p=0·045)	48·6% (23·1 to 65·7, p=0·0012)‡
Matched controls	21 714	235	65 214 756	0·36	Ref	Ref
**El Tor cholera outcomes by age**						
Age <5 years at selection						
El Tor cholera index cases	1340	17	4 074 969	0·42	21·6% (–58·6 to 52·2; p=0·34)	36·2% (–5·0 to 61·3, p=0·077)§
Matched controls	8040	133	24 930 109	0·53	Ref	Ref
Age ≥5 years at selection						
El Tor cholera index cases	2279	9	6 818 187	0·13	47·7% (–3·5 to 73·6; p=0·063)	61·7% (23·6 to 80·8, p=0·0065)¶
Matched controls	13 674	102	40 284 647	0·25	Ref	Ref
**El Tor cholera outcomes by homologous serotype**						
El Tor Inaba outcomes						
El Tor Inaba index cases	597	2	1 824 984	0·11	–50·1% (–503 to 62·6, p=0·57)	–51·6% (–513 to 62·6, p=0·56)||
Matched controls	3582	8	10 955 049	0·07	Ref	Ref
El Tor Ogawa outcomes						
El Tor Ogawa index cases	3022	19	9 068 172	0·21	32·0% (–8·4 to 57·4, p=0·11)	48·9% (17·9 to 68·2, p=0·0055)∗∗
Matched controls	18 132	168	54 259 707	0·31	Ref	Ref
**El Tor cholera outcomes by heterologous serotype**						
El Tor Ogawa outcomes						
El Tor Inaba index cases	597	2	1 824 984	0·11	51·9% (–105 to 88·7, p=0·32)	58·9% (–79·7 to 90·6, p=0·24)††
Matched controls	3582	25	10 955 049	0·23	Ref	Ref
El Tor Inaba outcomes						
El Tor Ogawa index cases	3022	3	9 068 172	0·03	47·0% (–72·5 to 83·7, p=0·29)	58·8% (–35·8 to 87·5, p=0·15)‡‡
Matched controls	18 132	34	54 259 707	0·06	Ref	Ref

∗ Adjusted for the matching variable (age at selection), as well as selected demographic variables known to be associated with the risk of treated cholera in Matlab: overall outcomes as well as El Tor cholera outcomes by age and homologous serotype were adjusted for distance from residence to the Dhonagoda River and distance from residence to hospital, while El Tor cholera outcomes by heterologous serotype were adjusted for distance from residence to hospital.

† Goodness of fit test (Score test statistic=135·0, p<0·0001).

‡ Goodness of fit test (Score test statistic=116·6, p<0·0001).

§ Goodness of fit test (Score test statistic=56·2, p<0·0001).

¶ Goodness of fit test (Score test statistic=55·5, p<0·0001).

|| No covariate.

∗∗ Goodness of fit test (Score test statistic=86·8, p<0·0001).

†† Goodness of fit test (Score test statistic=13·8, p=0·0032).

‡‡ Goodness of fit test (Score test statistic=15·0, p=0·0018)

Because relatively few index patients had classical biotype cholera (n=306 [7·8%]) and because the classical biotype is no longer circulating globally, it was of interest to explore the relationship further for protection against El Tor cholera associated with earlier El Tor index episodes (table 2). 26 episodes of El Tor cholera were observed among 3619 index patients with El Tor cholera (0·24 episodes per 100 000 person-days) as opposed to 235 episodes of cholera among 21 714 matched controls (0·36 episodes per 100 000 person-days; unadjusted PE 33·6% [95% CI 0·8–55·5], p=0·045; adjusted PE 48·6%; [23·1–65·7], p=0·0012). Again, the curve for adjusted instantaneous protection showed less than 5% decline over 10 years, and the drop was not significant (p=0·87; figure 3B).

**Figure 4:**
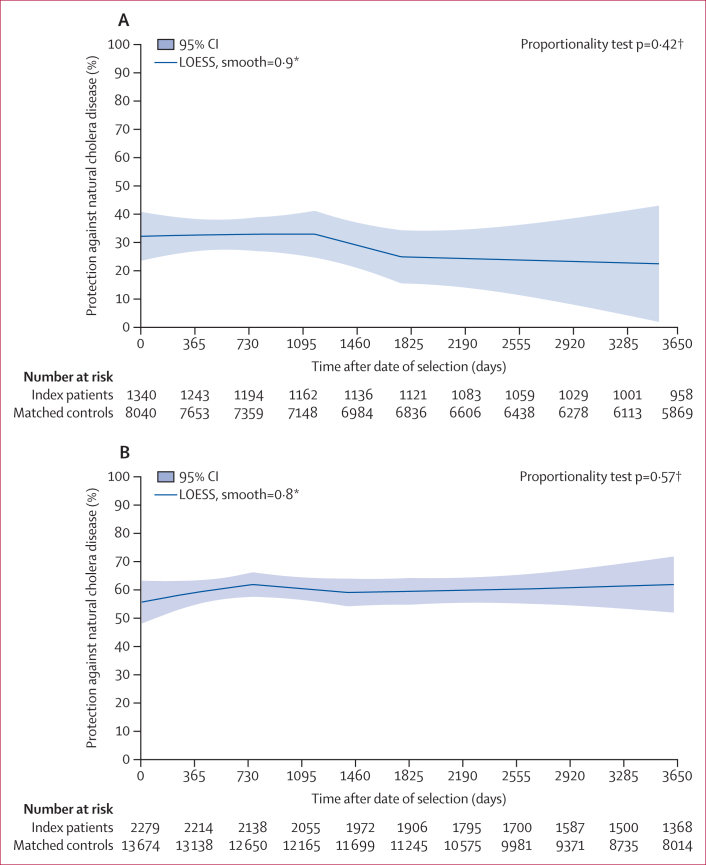
Smoothed curves for adjusted instantaneous protective associations between index and subsequent El Tor cholera by age at selection <5 years (A) and age at selection ≥5 years (B) Associations were adjusted for the matching variable (age at selection), as well as selected demographic variables known to be associated with the risk of treated cholera in Matlab, including distance from residence to the Dhonagoda River and distance from residence to hospital. ∗LOESS (locally estimated scatterplot smoothing), a non-parametric technique that uses local weighted regression to fit a smooth curve through points in a scatter plot, is described in the Methods. Smooth=0·9 (A) and 0·8 (B), which are optimally chosen values for the smoothing parameter, which ranges from 0·1 to 0·9 using the bias-corrected Akaike information criterion. The figure presents the fitted localised regression curve using 90% (A) and 80% (B) neighbouring data points. †Proportionality test for waning of protection as described in the Methods.

When protective associations between index and subsequent El Tor episodes were analysed by age on date of selection (table 2), there were 17 episodes among 1340 index patients selected at ages younger than 5 years (0·42 episodes per 100 000 person-days) versus 133 episodes among 8040 age-matched controls (0·53 episodes per 100 000 person-days; unadjusted PE 21·6% [95% CI –58·6 to 52·2], p=0·34; adjusted PE 36·2% [–5·0 to 61·3], p=0·077). For individuals aged 5 years and older, the comparative incidence (nine episodes among 2279 index patients, 0·13 episodes per 100 000 person-days; 102 episodes among 13 674 controls, 0·25 episodes per 100 000 person-days) yielded an unadjusted PE of 47·7% (–3·5 to 73·6, p=0·063) and an adjusted PE of 61·7% (23·6 to 80·8, p=0·0065). The difference in protective associations by age group was not significant (p=0·26). The smoothed curve for adjusted instantaneous protection in the younger age group decreased by only approximately 5% during the 10-year period of follow-up (figure 4A), and this decrease was not significant (p=0·42). The curve for the older age group was nearly constant over time, with no statistical evidence of waning (p=0·57; figure 4B).

As shown in table 2, too few subsequent El Tor Inaba episodes occurred in El Tor Inaba index patients and their controls for a meaningful assessment of serotype-homologous protection associated with the El Tor Inaba serotype. The incidence of subsequent El Tor Ogawa cholera among El Tor Ogawa index patients (19 episodes among 3022 index patients, 0·21 episodes per 100 000 person-days) and their controls (168 episodes among 18 132 controls, 0·31 episodes per 100 000 person-days) gave an unadjusted estimate of PE associated with the El Tor Ogawa serotype of 32·0% (95% CI –8·4 to 57·4, p=0·11) and an adjusted PE of 48·9% (17·9 to 68·2, p=0·0055), with no evidence of waning over the follow-up period (appendix p 1).

Estimates of El Tor serotype-heterologous protection were imprecise due to a modest number of post-selection cholera episodes among index patients and their controls (table 2). However, point estimates of unadjusted (PE 51·9%, [95% CI –105 to 88·7], p=0·32; PE 47·0% [–72·5 to 83·7], p=0·29) and adjusted (PE 58·9% [–79·7 to 90·6], p=0·24; PE 58·8% [–35·8 to 87·5], p=0·15) protection associated with El Tor Inaba and El Tor Ogawa index episodes, respectively, were comparable in magnitude. As shown in the appendix (pp 2–3), protection showed suggestive evidence of waning for El Tor Ogawa index cases and very little evidence of waning among El Tor Inaba index patients, with neither trend reaching statistical significance.

## Discussion

Our findings indicate that individuals who had been treated for cholera due to *V cholerae* O1 during the period 1990–2009 were at 50·2% reduced risk of a subsequent, treated serogroup O1 cholera episode during up to 10 years of follow-up that extended to 2014, and that protection associated with index episodes was stable during the entire period of follow-up. Restriction of this analysis to El Tor biotype cholera, the current pandemic biotype circulating globally, revealed a similar level of protection (48·6%). These findings represent, to our knowledge, the first assessment of long-term natural protection associated with cholera.

Children younger than 5 years at the time of an initial El Tor cholera episode exhibited a lower level of protection (36·2%) than individuals aged 5 years or older with initial El Tor cholera (61·7%). Although this difference was not statistically significant (p=0·26), it is interesting that the lower level of protection observed in younger children parallels the age-specific pattern of protection for currently used inactivated OCVs.^7^^,^^18^ Protection in young children appeared to decline slightly over 10-year follow-up, but this decline was not significant (p=0·42). There was no evidence of decline in the older age group. Despite the scale of our study, we lacked adequate statistical power to fully evaluate the effect of serotype on protection associated with El Tor cholera, although El Tor Ogawa was associated with significant (48·9%) protection against homologous disease that was sustained for the 10-year period of follow-up, and point estimates of protection associated with El Tor Inaba and Ogawa cholera against serotype-heterologous El Tor cholera (58·9% and 58·8%, respectively) suggested moderate protection by both.

It is important to acknowledge that our analysis had several limitations. First, it would seem plausible that individuals who were identified as index patients were at inherently higher risk of cholera than their matched controls, and might also have had a greater tendency to seek care for diarrhoea, biases that would have rendered our estimates of protection conservative. Second, our study assessed protective associations only for symptomatic cases of cholera severe enough to warrant solicitation of treatment. Greater immune responses to cholera are seen with severe than non-severe cholera. Therefore, it cannot be assumed that our findings pertain to *V cholerae* infections per se, the majority of which are mild or asymptomatic. Third, our findings were derived from a population with highly endemic cholera. This means that the population likely had previous *V cholerae* infections before their selection in the study and that, after selection, additional undetected infections occurred that could have boosted or maintained the immune response induced by index infections and generated immune responses in the matched controls. While OCV vaccine trials have identified the importance of immunological memory in maintaining protection against cholera, our findings should not be interpreted as maintenance of memory responses to the initially detected *V cholerae* infections per se as the basis for the long-term protection observed in this study.^19^^,^^20^ Therefore, our findings cannot be generalised to populations without endemic cholera. Fourth, the statistical tests that we used to detect waning of PE might not have had adequate power to detect modest degrees of waning. Finally, it might be argued that our findings might have been affected by an earlier trial of inactivated OCVs initiated in Matlab in 1985. However, at 5 years of follow-up of the trial, when enrolment of index patients for the present study began, no residual protection against cholera by these vaccines was evident.^12^

Several past studies have evaluated the protective associations between initial and subsequent episodes of cholera in populations with endemic cholera.^3^, ^4^, ^5^^,^^21^ All of these studies have been conducted in Matlab, Bangladesh, due to its unique longitudinal surveillance system in a large population. Although one study failed to find protection, likely due to the inclusion of mild cholera cases detected in active household surveillance, all other studies found protective relationships between initial and subsequent cholera episodes severe enough to be detected in treatment settings.^21^ Only one previous study was adequately powered to evaluate protection by El Tor cholera against subsequent El Tor cholera.^5^ This earlier analysis found that protection was 65% for 3 years after initial El Tor cholera cases, quite similar to our estimate of 48·6%. In contrast to the present analysis, however, the earlier analysis found similar point estimates of protection in children younger than 5 years versus individuals aged 5 years and older at the time that index cholera episodes were selected. As noted earlier, however, the differences of protection by age observed in our analysis could have arisen by chance. Moreover, the present analysis differs from this earlier analysis in its longer period and different secular interval of enrolment of index patients, in its more stringent exclusion of individuals with past histories of cholera, in its larger sample size, and in its much longer period of post-selection follow-up (10 years *vs* 3 years).

There has been evidence of serotype-homologous protection by both Ogawa and Inaba monovalent parenteral vaccines evaluated in past vaccine trials in Matlab. Furthermore, suggestive evidence from these trials indicated that heterologous protection against Ogawa cholera is conferred by Inaba lipopolysaccharide (LPS) O1 antigen, whereas protection by Ogawa LPS O antigen is only homologous.^22^ A previously published analysis of the risk of subsequent cholera after an initial episode supported these relationships, for which a biological basis is not clear.^5^ By contrast, the present analysis showed equivalent serotype-heterologous protection by El Tor Ogawa and Inaba cholera episodes, albeit with imprecise estimates of protection due to the limited statistical power. As this issue has clear relevance for the design of future cholera vaccines, it requires additional study.

Our findings will help inform the development of dynamic, population-based, transmission models of endemic cholera that can be used to explain and predict trends in cholera incidence and to analyse the long-term protective impact of currently deployed inactivated OCVs. The findings could also have several implications for present and future OCVs intended for use in populations with endemic cholera. Overall protection associated with naturally occurring El Tor cholera against subsequent cholera, although sustained regardless of the age of the initial episode, was moderate in magnitude, and protection associated with cholera in young children was suggestively lower than for older individuals, patterns seen with currently licensed, inactivated OCVs. These observations suggest that, unless future vaccines can elicit greater protective immunity against cholera than that provided by natural cholera, the upper limit of protection attainable with these vaccines might be moderate, although as demonstrated elsewhere, their population-level impact can be greatly augmented by vaccine herd protection.^23^ Finally, the lack of waning of protection over 10 years of follow-up, while likely due to the combined effects of immune responses to initial treated cholera disease followed by immunological boosting by subsequent *V cholerae* infections, may indicate that long-term protection by OCVs might be attainable in populations with endemic cholera, a prediction supported by the finding of long-term protection of older age groups by currently deployed, inactivated OCVs.^7^

## Data sharing

Data used for the analyses in this paper will be made available to all interested researchers following the published policies of the icddr,b and the International Vaccine Institute.

## Declaration of interests

We declare no competing interests.
