# Supplementary material for: Protection by natural cholera against later episodes of cholera over 10 years of follow-up in Matlab, Bangladesh: a retrospective cohort study
Source: Lancet Microbe. 2025 Mar;6(3):None. doi: 10.1016/j.lanmic.2024.100981 (PMC11876100; doi:10.1016/j.lanmic.2024.100981)
Supplement: Supplementary appendix [file mmc1.pdf]

THE LANCET
Microbe

Supplementary appendix

This appendix formed part of the original submission and has been peer reviewed.
We post it as supplied by the authors.

Supplement to: Hoque M, Kim DR, Ahmmed F, et al. Protection by natural cholera against later episodes of cholera over 10 years of follow-up in Matlab, Bangladesh: a retrospective cohort study. *Lancet Microbe* 2025. https://doi.org/10.1016/j.lanmic.2024.100981

Supplementary Figure 1. Smoothed curves for adjusted* instantaneous protective associations between index and subsequent serotype homologous, El Tor Ogawa cholera episodes

*Associations were adjusted for the matching variable (age at selection), as well as selected demographic variables known to be associated with the risk of treated cholera in Matlab, including distance from residence to the Dhonagoda River, and distance from residence to hospital.

+ LOESS (locally estimated scatterplot smoothing), a nonparametric technique that uses local weighted regression to fit a smooth curve through points in a scatter plot, is described in the Methods. Smooth=0.9 which is an optimally chosen smoothing value for the parameter, which ranges from 0.1 to 0.9 using bias-corrected Akaike information criterion. The figure presents the fitted localized regression curve using 90% neighboring data points.

‡ Proportionality test for waning of protection as described in the Methods.

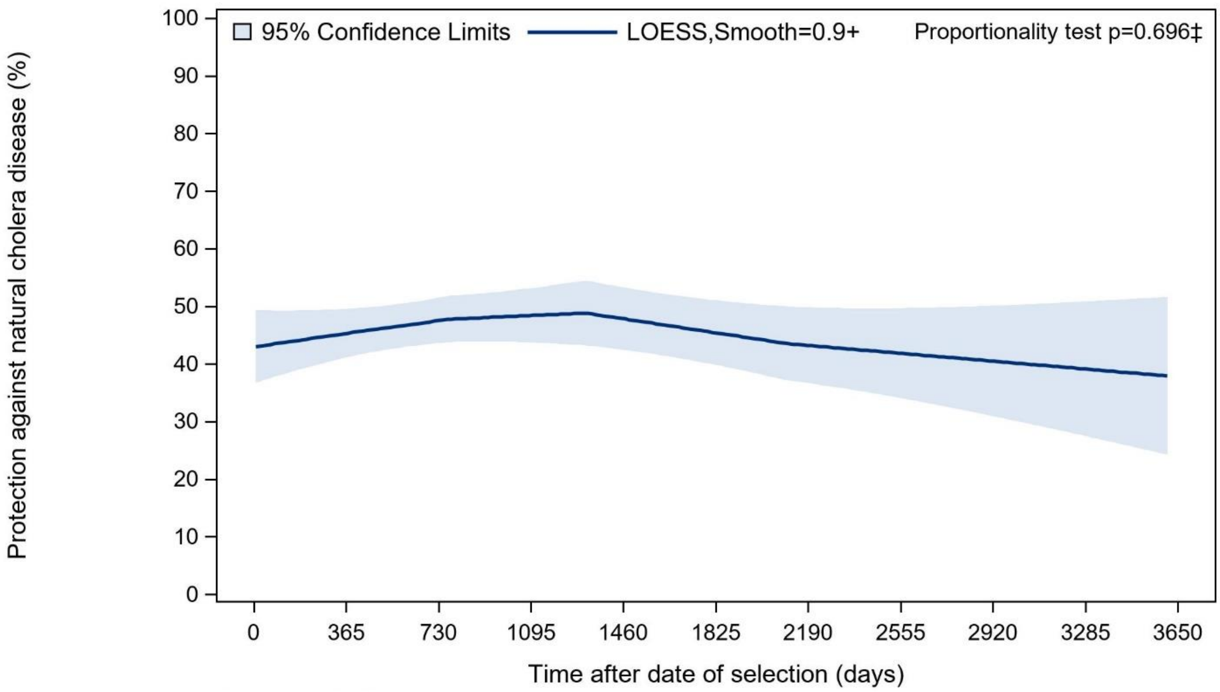

Number at Risk

**Index Patients**	3022	2885	2781	2680	2597	2525	2393	2289	2167	2077	1922
**Matched Controls**	18132	17361	16696	16099	15589	15079	14278	13624	12960	12280	11430

Supplementary Figure 2A and 2B. Smoothed curves for adjusted* instantaneous protective associations between index and subsequent serotype heterologous: El Tor Inaba index against El Tor Ogawa episodes (A) and El Tor Ogawa index against El Tor Inaba episodes (B)

*Associations were adjusted for the matching variable (age at selection), as well as selected demographic variables known to be associated with the risk of treated cholera in Matlab, including distance from residence to hospital (A and B).

†LOESS (locally estimated scatterplot smoothing), a nonparametric technique that uses local weighted regression to fit a smooth curve through points in a scatter plot, is described in the Methods. Smooth=0.9 (A) and 0.9 (B), which are optimally chosen values for the smoothing parameter, which ranges from 0.1 to 0.9 using the bias-corrected Akaike information criterion. The figure presents the fitted localized regression curve using 90% (a and b) neighboring data points.

‡Proportionality test for waning of protection as described in the Methods.

(A)

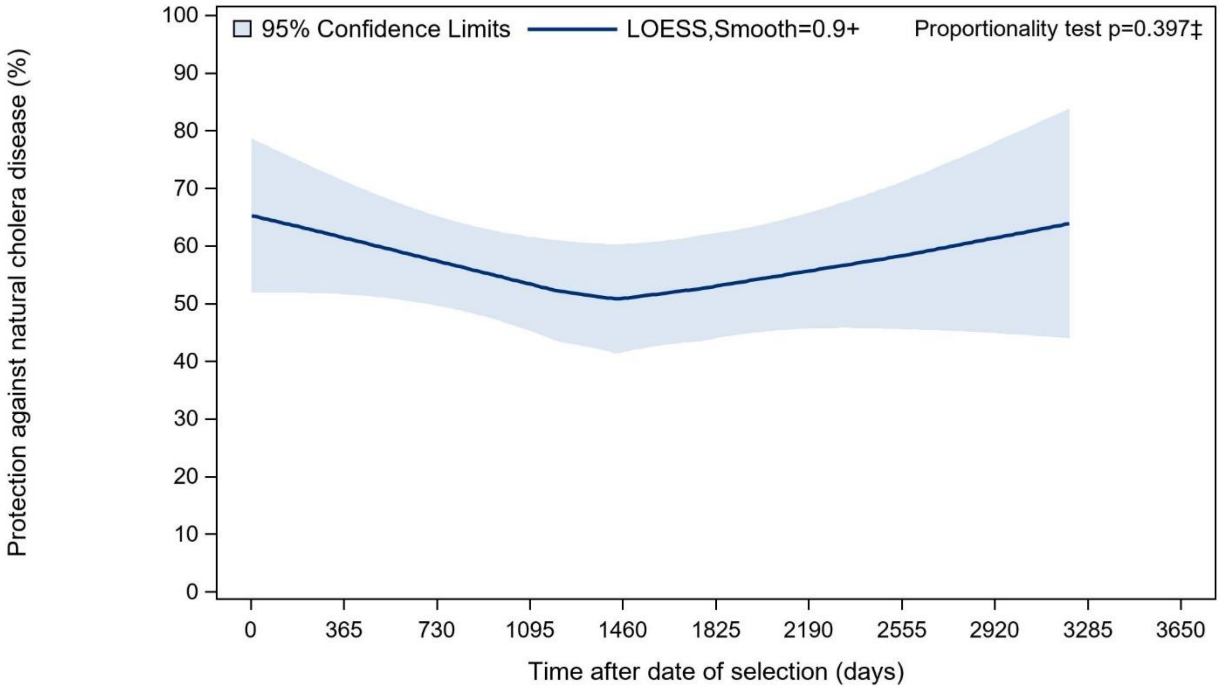

Number at Risk

**Index Patients**	597	572	551	537	511	502	485	470	449	424	404
**Matched Controls**	3582	3430	3313	3214	3094	3002	2903	2795	2689	2568	2453

(B)

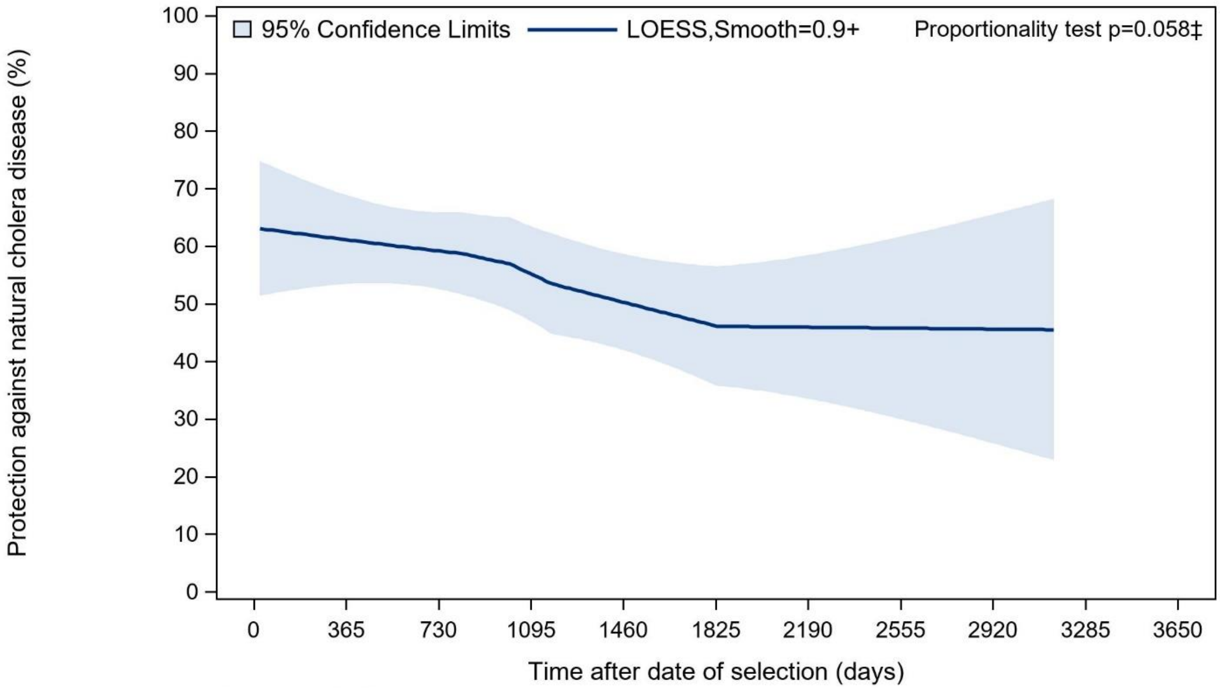

Number at Risk

**Index Patients**	3022	2885	2781	2680	2597	2525	2393	2289	2167	2077	1922
**Matched Controls**	18132	17361	16696	16099	15589	15079	14278	13624	12960	12280	11430
